# Actinomycosis of the Appendix in Childhood- An Unusual Cause of Appendicitis

**Published:** 2014-09-01

**Authors:** Esra Karakus, Ervin Mambet, Müjdem Nur Azılı, Belgin Gülhan, Tuğrul Tiryaki, Hasan Tezer

**Affiliations:** 1 Department of Pathology, Ankara Children's Hematology and Oncology Research and Training Hospital, Turkey; 2 Department of Pediatric Surgery, Ankara Children's Hematology and Oncology Research and Training Hospital, Turkey; 3 Department of Pediatrics, Ankara Children's Hematology and Oncology Research and Training Hospital, Turkey

**Keywords:** Appendicitis, Actinomycosis, Diagnosis

## Abstract

Actinomycosis is a rare chronic bacterial infectious disease in childhood. A 14-year-old boy admitted with cramping abdominal pain and vomiting. Physical examination revealed right lower quadrant tenderness. Appendectomy was performed. On the histological section, typical actinomycotic (sulfur) granules in the appendiceal lumen were observed.

## INTRODUCTION

Actinomycosis is a rare chronic bacterial infectious disease in children and is characterized by multiple abscesses, fistulae, draining sinuses, granulation, and fibrous tissue. It is caused by Actinomyces species which was first described in 1878 by Israel. [1]

Actinomycetes are filamentous, anaerobic, gram positive bacteria and are commensals of the oral and cervicofacial regions, respiratory tract,female genital organ, and gastrointestinal system.[2]In children, appendiceal actinomycosis with acute, perforated appendicitis is quite rare.[3]

## CASE REPORT

A 14-year-old boy was admitted with cramping abdominal pain and vomiting. Physical examination revealed right lower quadrant rebound tenderness. The white blood cell was 10,400/ul and serum C-reactive protein was increased at 3.56mg/dl. Increased (10mm) thickness of the appendix with nonperistaltic ileocecal region was noted by abdominal ultrasound (US). After preoperative preperation, appendectomy was performed. Grossly inflammed and enlarged appendix was noted. The pathologic specimen consisted of a vermiform appendix measuring 8cm x 3.5cm in dimension. The lumen contained a fecalith. The sections revealed acute inflamation. The inflammatory reaction was predominantly neutrophilic. With higher magnification typical actinomycotic (sulfur) granules in the appendiceal lumen were observed which were confirmed as appendiceal actinomycosis (Fig. 1). Stains for other organisms including acid-fast basilli and fungi were negative. The appendix wall was in suppurative stage. The patient did well postoperatively.

**Figure:**
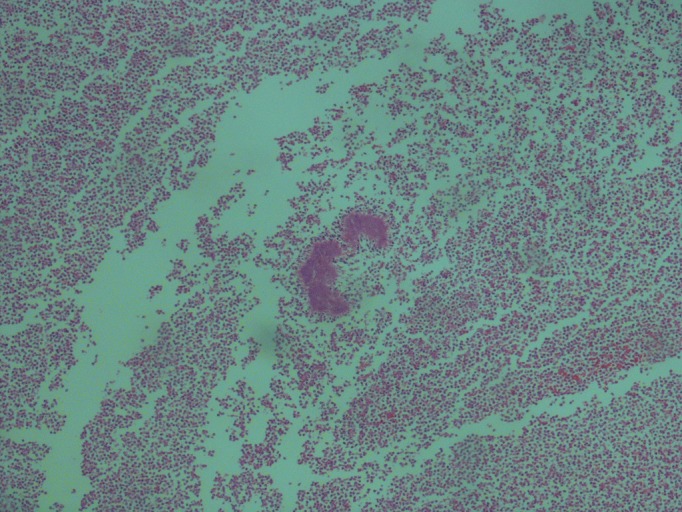
Figure 1:Histologic specimen showing sulfur granule.

## DISCUSSION

Actinomycosis is an uncommon disease.Actinomyces israelii is the most common cause of the Actinomycosis in humans.[4] Preoperative diagnosis of the Actinomycosis is a rare and difficult. In abdominal actinomycosis, the appendix and ileocecal regions are usually involved. Factors that predispose to abdominal actinomycosis include abdominal surgery, trauma, neoplasia, foreign bodies, and bowel perforation. Other predisposing factors are immunsuppression, malnutrition, and human immunodeficiency virus infections. Appendiceal actinomycosis is however rare in childtren. Abdominal actinomycosis may lead to nonspecific findings including mass, abscesses and sinuses and may be confused with diseases like diverticulitis, Crohn’s disease, ulcerative colitis and tubo-ovarian abscesses. The extensive fibrosis of actinomycotic lesions that appear as solid nodular or mass lesions are indistinguishable from malignancies. Sinus formation involving the perianal region can mimic Crohn's disease or tuberculosis. There are no spesific radiological features for actinomycosis. Hematological and biochemical markers are also nonspecific.[1,2]

The infection progresses insidiously and spreads along contiguous organs, especially the liver and may even involve the retroperitoneal tissues, spine and the abdominal wall. It rarely spreads by hematogenous or lymphatic dissemination. In our patient, actinomycosis was limited to the appendix with no evidence of distant organ involvement.[3]

Antibiotic treatment consists of high doses of penicilin for four weeks. Treatment is continued for one year. Alternative first-line antibiotics include amoxicillin, tetracycline, erythomycin, and clindamycin.[4]To conclude, appendiceal actnimycosis is a rare pathology in children and should be listed in etiology of appendicitis.

## Footnotes

**Source of Support:** Nil

**Conflict of Interest:** None declared

